# Impact of Tributyltin (TBT) on Energy Metabolism and Redox Homeostasis in Blue Crab *Callinectes sapidus*

**DOI:** 10.3390/metabo15040253

**Published:** 2025-04-08

**Authors:** Leonardo Airton Ressel Simões, Rafaella Sanfelice Normann, Daniela Drosdowski, Bruna Selau, Marjoriane de Amaral, Alex Sander da Rosa Araujo, Anapaula Sommer Vinagre

**Affiliations:** 1 Comparative Metabolism and Endocrinology Laboratory (LAMEC), Federal University of Rio Grande do Sul (UFRGS), Porto Alegre 90035-003, RS, Brazil; 2 Cardiovascular Physiology Laboratory, Post Graduation Program in Biological Sciences: Physiology, Department of Physiology, Federal University of Rio Grande do Sul (UFRGS), Porto Alegre 90035-003, RS, Brazil

**Keywords:** crustaceans, antifouling, ecotoxicology, metabolism, oxidative stress

## Abstract

**Background/Objectives**: Tributyltin (TBT), a potent biocide used in antifouling paints, is highly toxic to aquatic environments, causing oxidative stress, endocrine dysfunction, and metabolic disorders in aquatic organisms, including crustaceans. The blue crab *Callinectes sapidus* is an economically and ecologically important species in several countries worldwide and is considered an invasive species in Europe. **Methods**: This study evaluated the effects of 7 days of exposure to environmentally relevant TBT concentrations (100 and 1000 ng.L^−1^) on the intermediary metabolism and oxidative balance of the blue crab *Callinectes sapidus*. After exposure, hemolymph samples were analyzed to determine glucose, lactate, total protein, and cholesterol levels. In tissue samples, concentrations of triglycerides, glycogen, total glutathione (GSH), reactive oxygen species (ROS), sulfhydryls, lipid peroxidation (LPO), and glutathione S-transferase (GST) activity were assessed. **Results**: In the hemolymph, glucose and lactate levels increased, while the total cholesterol, triglycerides, and total proteins decreased in all exposed groups. The GST activity increased in exposed tissues, while the total GSH and sulfhydryl content decreased. The ROS concentration increased in response to higher TBT concentrations. **Conclusions**: These findings highlight the toxicity of TBT and show that 7 days of exposure to environmentally relevant concentrations disrupts the metabolic homeostasis and oxidative balance in *C. sapidus*. Additionally, this study demonstrates that *C. sapidus* is sensitive to TBT exposure and has potential as a model species for ecotoxicological studies.

## 1. Introduction

The strong antifouling effect of organotin (OT) compounds was discovered in the 1950s, and from then until the mid-2000s, tributyltin (TBT) was widely used as a highly efficient biocide in antifouling paints on ship and boat hulls [1]. Although the use of TBT paints has been banned by the International Maritime Organization in 2008, its persistence in the environment and its rampant use have led to its widespread presence in water bodies, sediments, and even in organisms, threatening the aquatic environment [2]. Currently, TBT is often referred to as one of the most toxic anthropogenic chemicals which has been deliberately introduced into the environment in large quantities [1,3]. Furthermore, a recent study has shown that, despite being banned, TBT-based antifouling products continue to be produced, exported, imported, and marketed in Latin America and, possibly, in developed countries [4]. Recent studies show that TBT pollution has been incorrectly considered eradicated; therefore, research about the effects of TBT pollution on the aquatic environment and its organisms is still needed [5].

Reactive oxygen species (ROS), naturally synthesized inside mitochondria during aerobic respiration or as byproducts of phagocytosis, are important cellular messengers which are kept in redox homeostasis [6]. Redox imbalance occurs when the rate of ROS generation is higher than the rate of neutralization by antioxidant molecules and enzymes, promoting oxidation in lipids, proteins, and nucleic acids, affecting many metabolic and physiological functions [7,8]. TBT contamination has been associated with the increased production of ROS in many species of vertebrate and invertebrate animals, such as oysters and fishes [9,10,11,12]. Enzymatic and non-enzymatic antioxidant mechanisms are used by the animals as defense systems against ROS. The main antioxidant enzymes involved in maintaining the cellular redox balance are superoxide dismutase (SOD), catalase (CAT), and glutathione peroxidase (GPx) [13]. Glutathione S-transferase (GST), although not considered an antioxidant enzyme, plays an important role in redox homeostasis and in the biotransformation/elimination of ROS [14].

Marine crustaceans constitute a large group of animals, with more than 50,000 known species, which include many bioindicator species suitable for studies of the effects of contaminants such as TBT [1,15]. Decapod crustaceans play an important role in the transfer of nutrients and energy along the marine food chain and can transfer pollutants to other trophic levels [16]. In addition to the oxidative balance, the evaluation of the intermediary metabolism is also used in ecotoxicological studies with crustaceans and is useful to determine the health status of free-living animals [17]. With the analysis of these markers, it is possible to evaluate and observe the balance between the anabolic and catabolic pathways [18]. In crustaceans, for example, hyperlipidemic and hyperglycemic states are observed when the animal is exposed to different stress situations [19,20,21]. Hyperglycemia in crustaceans can be triggered by crustacean hyperglycemic hormone (CHH), a neurohormone secreted by the X organ–sinus gland (XO-SG) complex which is primarily involved in regulating glucose levels in the hemolymph [22,23]. In addition to hyperglycemia, CHH can also increase lipid levels in the hemolymph, playing a role in supplying glucose and lipids to meet the energy needs of the organs and tissues of decapod crustaceans [24].

*Callinectes sapidus*, popularly known as the blue crab, is a decapod species of great interest in ecotoxicological research due to its physiological characteristics, habits, and ease of handling, having been used in many experimental studies [25,26,27]. This decapod species occurs in the Atlantic Ocean, from Nova Scotia to northern Argentina, including the Gulf of Mexico [28]. It is also an important species in the fishing industry in the US, and for artisanal fishermen in Latin America, with a significant social and economic relevance [29]. In a previous study, the effects of the acute exposure to environmentally relevant concentrations of 100 or 1000 ng.L^−1^ TBT were evaluated in *C. sapidus*, and, after 96 h of exposure to 1000 ng.L^−1^ of TBT, the total proteins in hemolymph and cardiac glycogen increased [27]. Therefore, in this study, the crabs were exposed to the same TBT concentrations for a longer period (7 days) to investigate the effects of TBT exposure on the intermediary metabolism and oxidative balance.

## 2. Materials and Methods

### 2.1. Crab Collection, Maintenance, and Experimental Design

All experimental procedures performed followed current legislation and the standards of the Brazilian Institute of Environment and Renewable Natural Resources (IBAMA) for the capture and use of native invertebrates. The project has a license (ICMBio/SISBIO nº 81853-1) for the collection and use of decapod crustaceans in scientific research projects and provides annual reports to IBAMA regarding the number of animals captured.

Male crabs (*n* = 36) were collected from December 2022 to March 2023 on the east bank of Tramandaí Lagoon (29°59′26.1″ S 50°10′43.3″ W), in Tramandaí county, Rio Grande do Sul. This field site was previously analyzed and considered TBT free [27]. The animals were captured during the afternoon and evening (4:00 p.m. to 8:00 p.m.) during the summer season with a “coca” type fishing net baited for crustaceans. During collection, animals of the same size (length and width) were selected to ensure homogeneity of the life span among the animals. Four consecutive field excursions were necessary to obtain an adequate number “*n* = 9–12” of animals, estimated before the experiment begun. After collection, the animals were immediately transported in plastic boxes with local water and constant aeration to the Laboratory of Metabolism and Comparative Endocrinology (LAMEC) at the Federal University of Rio Grande do Sul (UFRGS), Porto Alegre, Brazil. In the laboratory, the animals were acclimated for one week in a 2000 L tank with constant aeration, 10‰ salinity, 24 ± 2 °C temperature, and natural photoperiod and fed with squid every two days. The water salinity for the acclimation period and during the experiment was defined in a previous study according to the local salinity values measured during expeditions [27].

After the acclimation period, the animals in the intermolt phase [30] were individually housed in 7.5 L aquariums containing 5 L of water with 10‰ salinity and constant aeration. The crabs were proportionally divided into three experimental groups and exposed to: control (water without TBT, *n* = 12), 100 ng.L^−1^ of TBT (*n* = 13), or 1000 ng.L^−1^ of TBT (*n* = 11) for 7 days. Animals were exposed to nominal concentrations in a semi-static system. The experiments were repeated 4 times with 3 replicates for each group (control and exposure). During the exposure period, the animals’ feeding was suspended and every 48 h the aquarium water was changed and a new TBT concentration was added. No deaths were registered during the exposure period. Tributyltin chloride (TBTCl, CAS No. 1461-22-9, with purity ≥ 97%) was purchased from Sigma-Aldrich (Barueri, São Paulo state, Brazil) and diluted in absolute ethanol (analytical grade > 99.5%) as the solvent vehicle to obtain the stock solution [31,32]. At the maximum concentration achieved (1000 ng.L^−1^), the volume of ethanol was 0.00278% (*v*/*v*), which, according to previous studies, has no harmful effect on small in-vertebrates, including crustaceans [31,32]. Therefore, it was not necessary to have a solvent control group. All chemical reagents used in this study are of analytical grade.

After the exposure period, the crabs were cryoeuthanized for 15 min. The morphometric data of the animals were verified, with the average weight of the crabs being 94 ± 4.73 g and a total length of 5.4 ± 0.11 cm and carapace width of 10.6 ± 0.23 cm (mean of the data ± standard error). Hemolymph samples were collected at the base of chelipeds with 1 mL disposable syringes with 10 µL of 8% potassium oxalate for every 100 µL of hemolymph to avoid coagulation. After collecting, the hemolymph samples were transferred to 1.5 mL microtubes with a conical bottom and frozen at −20 °C until the time of dosing. Tissue samples were collected from hepatopancreas, heart, cheliped muscles, and anterior and posterior gills. After sampling, the tissues were stored at −80 °C until used in the assays.

### 2.2. Biochemical Analysis

After thawing, hemolymph samples were centrifuged for five minutes at 2000 rpm to collect plasma samples used to determine metabolite concentrations. The concentrations of glucose, lactate, total proteins, total cholesterol, and triglycerides in the hemolymph were quantified by enzymatic methods with commercial kits (Labtest Diagnóstica SA, Lagoa Santa, Brazil) in 10 µL of sample, according to the manufacturer’s instructions. The samples were read in a spectrophotometer with specific wavelengths for each substrate: (1) glucose and triglycerides: 505 nm; (2) lactate: 550 nm; (3) total proteins: 545 nm; and (4) cholesterol: 500 nm. The results of glucose, triglycerides, lactate, and cholesterol were expressed in mg/dL of hemolymph and those of total proteins in g.dL^−1^ of hemolymph. The extraction and quantification of tissue glycogen was adapted from the techniques of Van Handel (1965) [33] and Geary et al. (1981) [34] by Inohara et al. (2015) [35]. The resulting glucose was quantified by the enzymatic glucose oxidase method with the same kit for hemolymphatic glucose. A glycogen standard curve (1 mg/mL) was used as a calibration factor and the results were expressed in mg glycogen.g tissue^−1^. Hepatopancreas and muscle samples used to determine triglyceride concentration were homogenized with 0.9% saline in a ratio of 10:1 (1 mg of tissue to 10 µL of saline) [36]. Samples (10 µL) of this suspension were mixed with the same enzymatic buffer used to determine hemolymph triglycerides. The results were expressed in mg triglycerides.g of tissue^−1^.

### 2.3. Oxidative Balance Analysis

For oxidative balance analysis, hepatopancreas, anterior and posterior gills, and muscles were homogenized in phosphate buffer (20 mM) with potassium chloride (140 mM) and 1 mM protease inhibitor (PMSF). Homogenization was performed at a ratio of 1 g of tissue: 5 mL of solution for gills and muscles and at a ratio of 1 g of tissue: 9 mL of solution for hepatopancreas. The homogenate obtained was centrifuged at 10,000× *g* for 10 min at 4 °C. Afterwards, the aliquots were separated into 0.5 mL microtubes and frozen at −20 °C for later analysis. Total protein concentration, lipid peroxidation levels (LPO), total reactive species content (Total ROS), sulfhydryls content, total glutathione (GSH), and glutathione s-transferase (GST) activity were analyzed in these supernatant samples.

Total protein concentration was measured by Lowry method [37]. Total ROS concentration was determined by the fluorescence method by reaction with dichlorofluorescein diacetate (DCFH-DA) (Sigma-Aldrich, Barueri, São Paulo state, Brazil), and the data were expressed in nmol.mg of protein^−1^ [38]. The amount of total sulfhydryl groups was determined according to Sedlak and Lindsay (1968) by the reaction of the thiol group with DTNB [39]. The sulfhydryls content groups was expressed as nmol TNB.mg of protein^−1^. Lipid peroxidation was measured by the reaction of oxidation products with thiobarbituric acid reactive substances (TBARSs), generating a colored compound, for which absorbance was read at 535 nm [40]. The results were represented as nmol.mg of protein^−1^. Glutathione S-transferase activity was measured according to the method of Boyland and Chasseaud (1969), by measuring the conjugation of 1-chloro2,4-dinitrobenzene (CDNB) with reduced glutathione (GSH) [41]. Activity was measured as the increase in absorbance values at 340 nm. Enzyme activity was expressed as nmol of CNDB conjugate.mg protein/min^−1^. For quantification of total GSH, samples were acidified with perchloric acid 2M to precipitate non-GSH sulfhydryl groups, and then the occurrence of GSH was measured with DTNB and expressed as nmol TNB.mg protein^−1^.

### 2.4. Statistical Analysis

The assessment of normal distribution was performed using the Shapiro–Wilk normality test. For variables with normal distribution, the ANOVA One Way test and Tukey’s post hoc test were used. For variables that did not present normal distribution, the Kruskal–Wallis test and Dunn’s post hoc test were used. In all analyses, the GraphPad Prism^®^ version 6.01 program was used and the significance and statistical power of 0.05 and 95% were established, respectively.

## 3. Results

### 3.1. Hemolymph Metabolites

**Figure 1:**
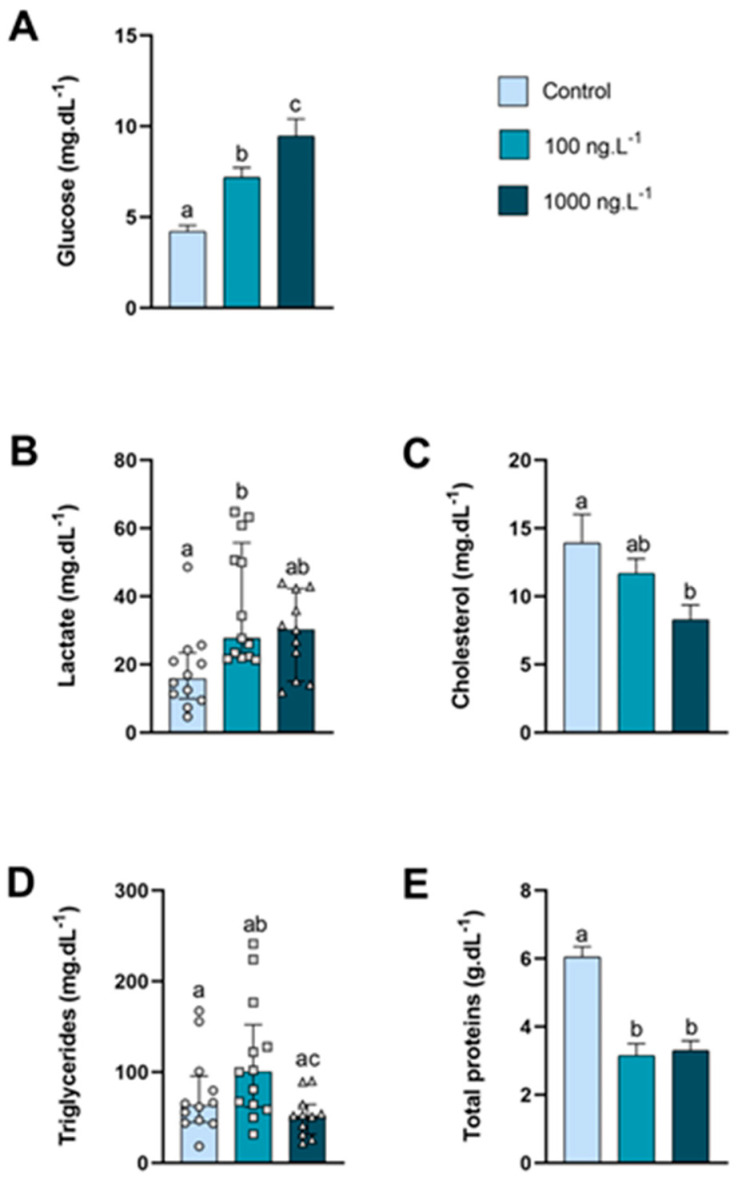
Metabolite levels in the hemolymph of *Callinectes sapidus* exposed to 100 or 1000 ng/L^−1^ of TBT for 7 days. Values represent the mean ± standard error (**A**,**C**,**E**) or median ± 10–90 interquartile range (**B**,**D**). Different letters indicate significant differences (*p* < 0.05) between experimental groups according to the ANOVA followed by Tukey’s post hoc test (parametric results) or the Kruskal–Wallis test followed by Dunn’s post hoc test (nonparametric results).

The glucose levels in the hemolymph (Figure 1A) of animals exposed to 100 ng.L^−1^ of TBT and 1000 ng.L^−1^ of TBT increased by 70% (*p* = 0.052) and 124% (*p* < 0.0001), respectively, compared to the control group and in the 1000 ng.L^−1^ group compared to the 100 ng/L^−1^ group (*p* = 0.04). The circulating lactate levels (Figure 1B) of the 100 ng.L^−1^ group increased significantly compared to the control group. Cholesterol levels in the hemolymph (Figure 1C) decreased as the concentration increased, and in the group exposed to 1000 ng.L^−1^ of TBT the concentration was 40% lower than the control group (*p =* 0.037). Although not significant (*p =* 0.360), triglyceride levels (Figure 1D) increased by 75% in the group exposed to 100 ng.L^−1^ compared to the control; however, in the group exposed to the 1000 ng.L^−1^, triglycerides the concentration reduced significantly (*p =* 0.015) compared to the 100 ng.L^−1^ group. In both groups exposed to TBT, total proteins (Figure 1E) reduced significantly compared to the control group (*p* < 0.0001).

### 3.2. Energetic Metabolism in Cardiac and Striated Muscles

Although not significant, the heart glycogen (Table 1) increased by 25% in the group exposed to 100 ng/L^−1^ of TBT. In the cheliped muscles, the glycogen and triglycerides concentration were not affected by the TBT exposure (Table 1).

### 3.3. The Energetic and Oxidative Metabolism in the Hepatopancreas

**Figure 2:**
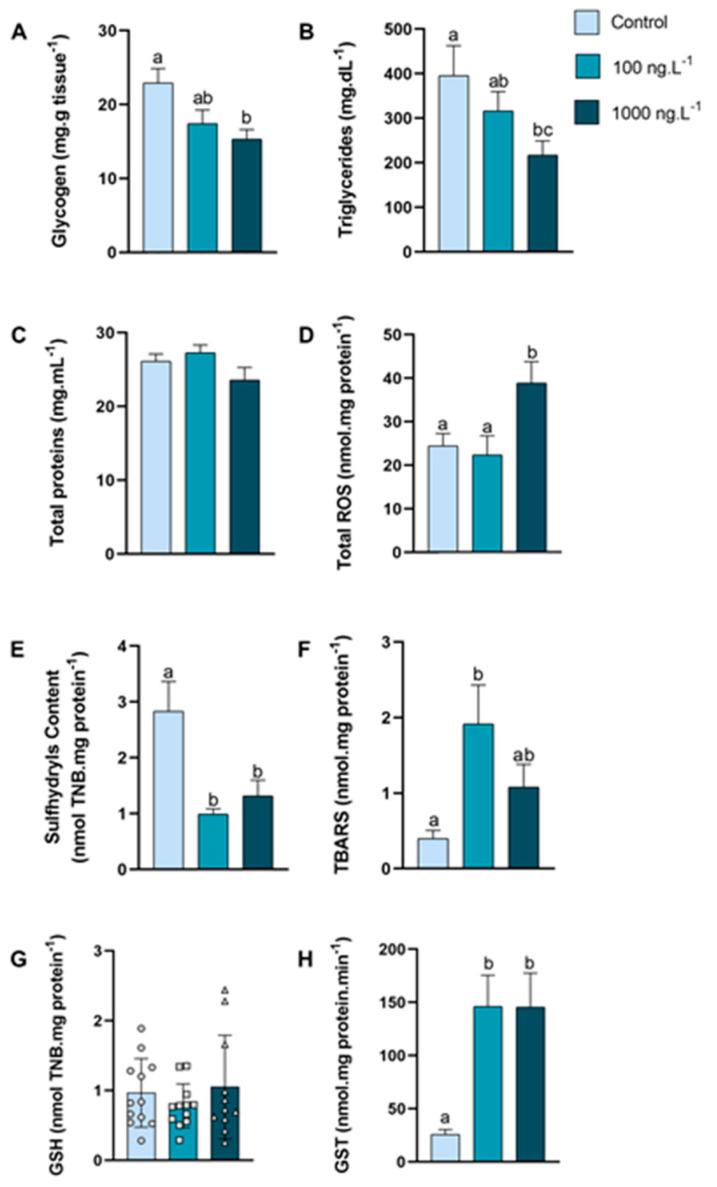
Concentration of metabolites and oxidative balance parameters in hepatopancreas of *Callinectes sapidus* exposed to 100 or 1000 ng.L^−1^ of TBT for 7 days. Values represent mean ± standard error (**A**–**F**,**H**) or median ± 10–90 interquartile range (**G**). ROS: reactive oxygen species. TBARS: Thiobarbituric acid reactive species. GSH: Reduced Glutathione. GST: Glutathione S-transferase activity. Different letters indicate significant differences (*p* < 0.05) between experimental groups according to ANOVA followed by Tukey’s post hoc test (parametric results) or Kruskal–Wallis test followed by Dunn’s post hoc test (nonparametric results).

In the hepatopancreas (Figure 2A), the glycogen concentration was reduced by 33% (*p =* 0.016) in the crabs exposed to 1000 ng.L^−1^ of TBT when compared to the control group. The triglycerides concentration (Figure 2B) decreased by 45% (*p =* 0.048) in the group exposed to 1000 ng.L^−1^ of TBT compared to the control group. The concentration of total proteins (Figure 2C) of the crabs did not show any significant difference.

The total reactive oxygen species (ROS) concentration (Figure 2D) of the crabs exposed to 1000 ng.L−1 of TBT increased by 59% (*p =* 0.021) when compared to the 100 ng.L^−1^ group and by 73% in comparison to the control group (*p =* 0.048). The sulfhydryls content (Figure 2E) was reduced by 65% in the group exposed to 100 ng.L^−1^ of TBT (*p =* 0.0023) and 53% in the group exposed to 1000 ng.L^−1^ (*p =* 0.0148) when compared with the control group. The lipid peroxidation, expressed as TBARs (Figure 2F), increased 389% (*p =* 0.0140) in the crabs exposed to 100 ng.L^−1^ of TBT when compared with the animals in the control group. GSH levels (Figure 2G) did not show significant differences between groups. The GST activity (Figure 2H) increased 466% in both groups (*p* = 0.0061 and *p* = 0.0089) exposed to TBT when compared to the control group.

### 3.4. The Energetic and Oxidative Metabolism in the Anterior Gills

**Figure 3:**
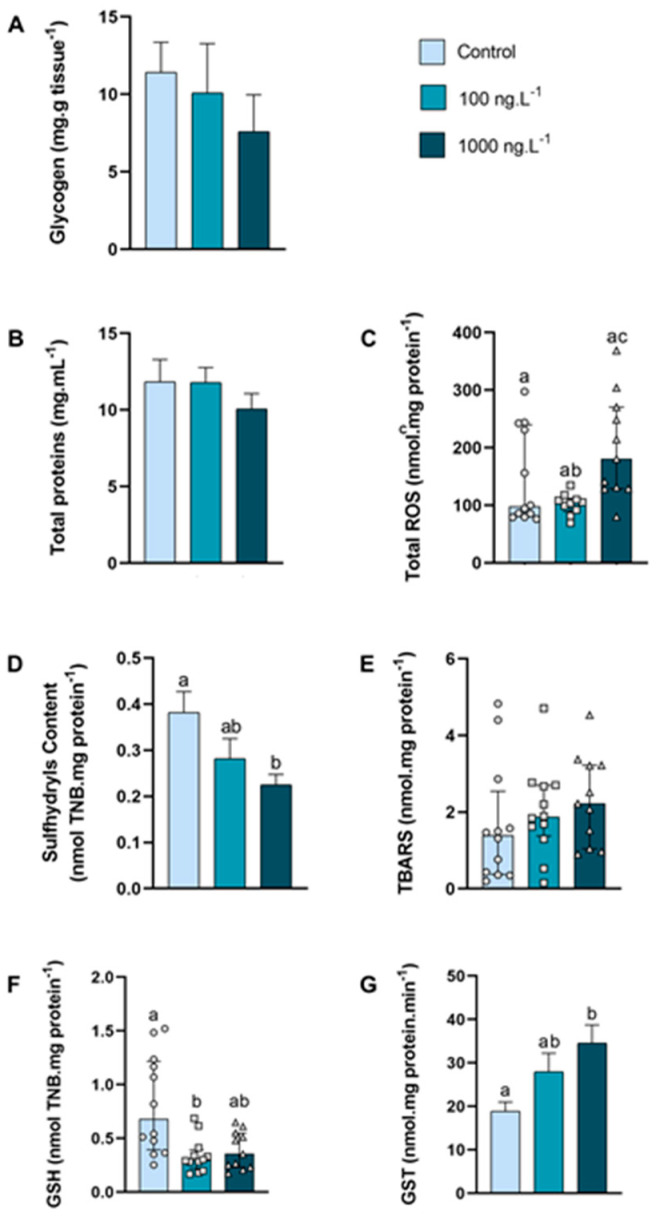
Concentration of metabolites and oxidative balance parameters in anterior gills of *Callinectes sapidus* exposed to 100 or 1000 ng/L^−1^ of TBT for 7 days. Values represent mean ± standard error (**A**,**B**,**D**,**G**) or median ± 10–90 interquartile range (**C**,**E**,**F**). ROS: reactive oxygen species. TBARS: Thiobarbituric acid reactive species. GSH: Reduced Glutathione. GST: Glutathione S-transferase activity. Different letters indicate significant differences (*p* < 0.05) between experimental groups according to ANOVA followed by Tukey’s post hoc test (parametric results) or Kruskal–Wallis test followed by Dunn’s post hoc test (nonparametric results).

In the anterior gills (Figure 3A), there was a tendency to decrease the glycogen concentration (*p* > 0.05) as the concentration of TBT increased, reaching 33% of the control values in the TBT 1000 ng.L^−1^ group. The concentration of total proteins (Figure 3B) of the crabs did not show any significant difference.

The total ROS concentration (Figure 3C) increased 95% (*p =* 0.026) in the 1000 ng.L^−1^ TBT group when compared to the 100 ng.L^−1^ group. The content of sulfhydryls (Figure 3D) of the 100 ng/L^−1^ group did not differ from the control group, while the sulfhydryls content of the 1000 ng.L^−1^ group reduced 42% (*p =* 0.0270) compared with the control group. The levels of lipid peroxidation (Figure 3E) did not differ between the groups. The total GSH levels (Figure 3F) reduced 58% (*p =* 0.0109) in the 100 ng.L^−1^ TBT group when compared with the control. The GST activity (Figure 3G) increased 82% (*p =* 0.0164) in the crabs exposed to 1000 ng.L^−1^ of TBT when compared to the control group.

### 3.5. Energetic and Oxidative Metabolism in Posterior Gills

**Figure 4:**
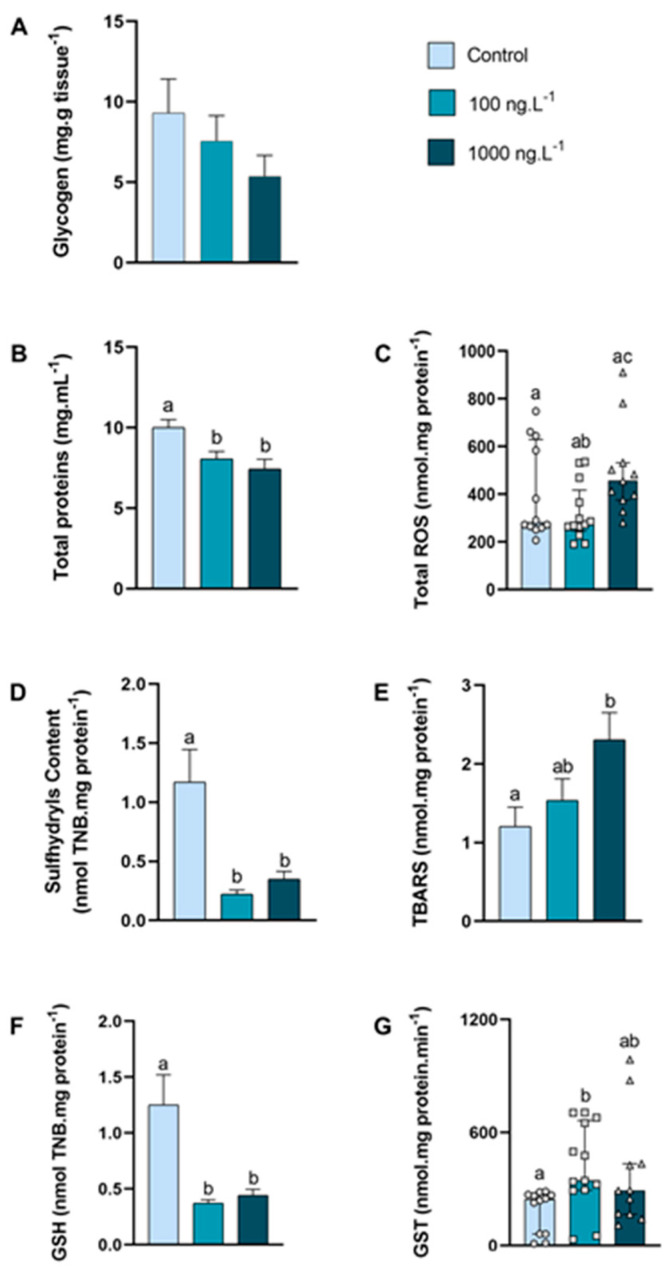
Concentration of metabolites and oxidative balance parameters in posterior gills of *Callinectes sapidus* exposed to 100 or 1000 ng/L^−1^ of TBT for 7 days. Values represent mean ± standard error (**A**,**B**,**D**,**E**,**F**) or median ± 10–90 interquartile range (**C**,**G**). ROS: reactive oxygen species. TBARS: Thiobarbituric acid reactive species. GSH: Reduced Glutathione. GST: Glutathione S-transferase activity. Different letters indicate significant differences (*p* < 0.05) between experimental groups according to ANOVA followed by Tukey’s post hoc test (parametric results) or Kruskal–Wallis test followed by Dunn’s post hoc test (nonparametric results).

In the posterior gills, the glycogen concentration (Figure 4A) tended to decrease as the concentration of TBT increased (*p* > 0.05), reaching 42% of the control values in the TBT 1000 ng.L^−1^ group. The concentration of total proteins (Figure 4B) was significantly reduced in both groups exposed to TBT compared to the control group (100 ng.L^−1^: *p =* 0.033; 1000 ng.L^−1^: *p =* 0.004).

The total ROS concentration (Figure 4C) increased 24% (*p =* 0.026) in the 1000 ng.L^−1^ TBT group when compared to the 100 ng.L^−1^ group. In both groups exposed to TBT, the sulfhydryls content (Figure 4D) reduced approximately 76% (*p =* 0.0007 and *p =* 0.0047) compared with the control group. The levels of lipid peroxidation (Figure 4E) increased 91% (*p =* 0.032) in the 1000 ng.L^−1^ TBT group when compared to the control group. The total GSH (Figure 4F) reduced approximately 70% in both groups exposed to TBT (100 ng.L^−1^: *p =* 0.0016 and 1000 ng.L^−1^: *p =* 0.0043), when compared with the control. The GST activity (Figure 4G) increased (*p =* 0.0071) in the crabs exposed to 100 ng.L^−1^ of TBT when compared to the control group. An interesting finding was that the GST activity in the posterior gills was approximately 3× higher than in the hepatopancreas and 12× higher than in the anterior gills.

## 4. Discussion

Considering that, despite its banishment, TBT is still registered in aquatic environments, and that its effects are not yet well elucidated in crustaceans, the evaluation of the acute and chronic effects of TBT in the laboratory is necessary to understand the toxic potential of this substance to these animals [27,42,43]. The TBT concentrations selected for the exposure of crabs were based on data indicating that the leaching of marine antifouling agents containing TBT compounds results in seawater concentrations ranging from 10 to 100 ng.L^−1^, with the highest levels recorded in estuaries, where aquatic organisms may be exposed to concentrations of up to 1.000 ng.L^−1^ [44]. Additionally, these concentrations have already been tested in previous studies and are considered sublethal, being widely used to assess the effects of TBT exposure in aquatic crustaceans [31]. In this study, no mortality was observed during *C. sapidus*’s exposure to these environmentally relevant concentrations of TBT. However, this does not rule out the possibility of mortality occurring with longer exposure periods.

In the present study, an increase in glucose and lactate was observed in the hemolymph of animals exposed to TBT for 7 days. In a previous study, when *C. sapidus* was exposed to the same TBT concentrations for 48 and 96 h, both metabolites tended to increase [27]. Glucose and lactate usually increase when animals are exposed to various types of stress, including anthropogenic actions [36] or exposure to xenobiotics [17]. An increase in the lactate concentration, the end-product anaerobic glycolysis, is normally related to glycogen mobilization during high energy demand or environmental hypoxia events [36,45,46,47]. It can also be intensified due to the exposure to contaminants, such as copper in *Carcinus maenas* [48]. Excess lactate is not easily excreted, instead it is sequestered in tissues, such as the hepatopancreas, to be converted back to glucose by the gluconeogenesis pathway [49]. In this study, the muscle glycogen was not affected by the TBT exposure, however the hepatopancreas glycogen decreased as TBT increased, which may explain the origin of both glucose and lactate in the TBT 1000 group. Therefore, increased glucose and lactate levels are a strong indication that the animals are stressed by TBT exposure.

The exposure to TBT resulted in decreased levels of cholesterol, triglycerides, and proteins in the hemolymph. Proteins play several roles, including energy supply, cellular structuring, catalyzing biological functions (enzymes), body defense (antibodies), hormones, and neurotransmitter synthesis [45]. The decrease in circulating proteins may be related to a possible uptake of amino acids by the tissues, specifically by the hepatopancreas and muscles, which can use alanine or glycine to produce glucose by the process of gluconeogenesis for energy production during stressful conditions [50,51,52]. In addition to lactate and amino acids, the glycerol produced by lipid digestion can also be converted to glucose by gluconeogenesis or to triglycerides by glyceroneogenesis [53], which can also explain the decrease in the lipids concentration in the hemolymph and hepatopancreas. The uptake of amino acids may also be necessary to cope with the energy expenditure when dealing with xenobiotics [54]. The total proteins content of the hepatopancreas, hemolymph, and ovary decreased when *Scylla serrata* was exposed to naphthalene, and this reduction may be attributed to the use of amino acids in several catabolic reactions [55]. In the crayfish *Astacus leptodactylus*, total proteins and cholesterol levels decreased significantly after seven days of exposure to glyphosate [17]. These authors also observed a significant increase in triglycerides and glucose levels, suggesting that sublethal concentrations of the xenobiotic may lead to the collapse of cellular homeostasis and alterations in the biochemical parameters of the hemolymph in this crayfish.

In the crayfish *Macrobrachium rosenbergii*, a chronic treatment (90 days) with TBT (10, 100, and 1000 ng.L^−1^) dissolved in water decreased the concentrations of glycogen, lipids, and proteins in the hepatopancreas [56]. After an acute exposure (24, 48, 72, and 96 h) to TBTCl dissolved in water, the glycogen concentration in the gonads, gills, muscle, and hepatopancreas of the crayfish *Macrobrachium kistnensis* gradually decreased [57]. In the present study, the glycogen concentration in the hepatopancreas of crabs exposed to the concentrations of 100 and 1000 ng.L^−1^ of TBT reduced after exposure for seven days. In both types of gills, although not significant, a tendency towards a concentration-dependent reduction in the glycogen concentration was observed. In the heart and skeletal muscles, the glycogen concentration did not change in the present study. In our previous study [27], glycogen levels in the heart increased after exposure to 100 and 1000 ng.L^−1^ of TBT for 96 h. It is likely that this increase observed after 96 h was recovered within 7 days. It is important to note that these results are the only data available regarding the effects of TBT on the hearts of crustaceans. Additionally, as observed in both the previous and the present study, no changes were detected in muscle glycogen levels following the exposure to TBT. Glycogen is the primary form of glucose storage in animals, including crustaceans. During periods of stress, such as exposure to xenobiotics, glycogen is broken down to provide glucose, which is rapidly taken up to meet energy demands [58]. Therefore, TBT, like other environmental stressors, can induce the stress response in crustaceans by increasing the activity of enzymes involved in glycogenolysis, especially in the hepatopancreas [59].

GST is an enzyme with an important function in the detoxification of organic contaminants and endogenous compounds because it catalyzes the conjugation of the reduced form of GSH to a xenobiotic, which is an important biomarker of the elimination process [60]. In addition, GST is important in the elimination of ROS [61]. The anterior gills showed increased GST activity when the animals were exposed to the highest concentration, while in the hepatopancreas, the exposure to both TBT concentrations increased GST activity. The exposure of the mollusk *Meretrix petechialis* to bisphenol A concentrations led to a dose-dependent increase in GST activity and malondialdehyde (MDA) content [62]. The exposure of the marine copepod *Tigriopus japonicus* to the antifouling biocide Diuron, increased intracellular ROS and lipoperoxidation levels, reduced GSH, and increased GST activity in the whole body [63]. The hepatopancreas is the main organ of detoxification and nutrient storage and, therefore, acts as an indicator of pollution [64,65]. The increase in the GST activity in the hepatopancreas may be related to its capacity for the biotransformation and detoxification of xenobiotics. Although the tissue presented an increase in total ROS when the animals were exposed to a concentration of 1000 ng.L^−1^, the increase in the GST activity may have been efficient in avoiding an increase in lipid peroxidation in this tissue. The same may have occurred in the anterior gills, which, even with an increase in total ROS production, did not show an increase in lipid peroxidation.

Considering that in crustaceans the anterior and posterior gills have different functions they were analyzed separately. Because they are constituted by a respiratory epithelium, the anterior gills are responsible for the respiratory gases exchange, while the posterior gills, formed by a thick epithelium rich in mitochondria, are the main sites of ion transport and, therefore, of osmoregulation processes [52,66,67]. It has been reported that organotins can trigger apoptosis through the mitochondrial pathway, blocking mitochondrial ATP synthesis, causing the loss of mitochondrial membrane integrity, increasing oxidative stress, and promoting ROS production [68]. The membrane degradation by lipid peroxidation, one of the main forms of damage caused by ROS, after the exposure to TBT induces a rapid loss of gill function [69].

In the present study, a concentration–response relationship was observed between the TBT concentration and TBARS content in the hepatopancreas and posterior gills. Under TBT-induced oxidative stress conditions, the levels of TBARS induction in the hepatopancreas increased significantly at a concentration of 100 ng/L^−1^, whereas in the posterior gills, it increased significantly at a concentration of 1000 ng/L^−1^. The lack of changes in TBARS levels in the anterior gills and the hepatopancreas at the highest concentration may be related to the total sulfhydryl reserves. These results suggest that the hepatopancreas of crabs is more vulnerable to lipid peroxidation than the anterior gills. Although a tendency towards an increased lipid peroxidation was observed in the anterior gills, the posterior gills showed an increase in LPO at the 1000 ng/L^−1^ concentration of TBT, and cellular damage was shown. These results may indicate that crustacean’s gills are the first targets of TBT contamination due to their direct contact with the external environment, causing damage to their physiological functions [70]. Meanwhile, the increase in TBARS levels in the posterior gills may be associated with the reduction in GSH and sulfhydryl levels in this tissue. A similar response pattern was observed in a study conducted with the crab *Eriocheir sinensis* when exposed to butachlor, an herbicide from the acetanilide class.

Lipid peroxidation damage occurs due to the reaction of ROS with the cell membrane, and in the present study, a significant increase in total ROS was observed in the hepatopancreas and anterior gills of crabs when exposed to the highest concentration of TBT. When the worm *Caenorhabditis elegans* was exposed to different concentrations of TBT, a dose-dependent increase in the total ROS was observed [71]. This increase in the total ROS was also reported in *Tigriopus japonicus* when exposed to all concentrations of triphenyltin (TPT) exposure. TPT is a chemical that has entered aquatic ecosystems, especially in the marine environment, due to its wide application in antifouling systems in ships and mariculture facilities [72].

Finally, GSH is a crucial non-enzymatic antioxidant in living organisms, effectively neutralizing free radicals, and represents the first line of defense against ROS [73,74]. Reduced glutathione plays a key role in the detoxification of a large number of xenobiotics and has been reported together with other antioxidant enzymes in marine invertebrates as part of antioxidant defenses [75]. GSH levels in the hepatopancreas remained similar, while in the anterior and posterior gills they were reduced in both TBT concentrations. In the gills of E. sinensis, the exposure to butachlor gradually decreased GSH levels as the contaminant concentration increased [76]. When GSH was evaluated in hemocytes of the crab *Scylla paramamosain* exposed to phenanthrene, one of the most abundant polycyclic aromatic hydrocarbons (PAHs) in the marine environment, its levels increased at the lowest exposure concentration, while at higher concentrations levels it remained like the control [77]. The literature suggests that the lower GSH levels in invertebrates exposed to TBT can be explained by the conjugation of GSH with the antifouling agent, which could imply that after prolonged environmental exposure, the animal could face a depletion of GSH and a subsequent reduction in their detoxification capacity, making them more vulnerable to oxidative stress [78,79].

## 5. Conclusions

In our study, exposure to TBT for a period of 7 days at environmentally relevant concentrations was able to alter the metabolic and oxidative homeostasis of the crab *C. sapidus*. These results highlight the potential of this species to be used as a model organism in ecotoxicological studies, given its sensitivity to this pollutant and its ecological relevance. The biological responses observed under laboratory exposure conditions reinforce the suitability of *C. sapidus* for assessing the impact of TBT contamination in aquatic environments and possibly other environmental pollutants. The conservation of *C. sapidus* populations and the maintenance of the aquatic ecosystem require the strict control of pollutants, such as TBT, and the implementation of effective strategies to reduce contamination. Understanding the effects of TBT and other pollutants is crucial to the development of conservation measures and environmental policies that protect not only the species in question but also the health of aquatic ecosystems as a whole.

## Figures and Tables

**Table 1:** Metabolites concentration in different types of muscles of *Callinectes sapidus* exposed to 100 or 1000 ng/L^−1^ of TBT for 7 days.

Tissue/Metabolite	Control	100 ng/L^−1^	1000 ng/L^−1^	*p*
Heart Muscle/Glycogen	50.04 ± 12.96	71.49 ± 17.76	49.47 ± 11.05	0.4751
Skeletal Muscle/Glycogen	3.59 ± 3.68	3.57 ± 3.75	3.21 ± 3.21	0.9730
Skeletal Muscle/Triglycerides	5.58 ± 1.96	5.47 ± 2.06	5.00 ± 1.55	0.7389

Values represent mean ± standard deviation.

## Data Availability

The original contributions presented in this study are included in the article. Further inquiries can be directed to the corresponding author.

## Abbreviations

OT	Organotin
TBT	Tributyltin
ROS	Reactive oxygen species
SOD	Superoxide dismutase
CAT	Catalase
GPx	Glutathione peroxidase
GST	Glutathione S-transferase
CHH	Crustacean hyperglycemic hormone
XO-SG	X organ–sinus gland
LPO	Lipid peroxidation
Total ROS	Total reactive species
GSH	Glutathione
DCFH-DA	Dichlorofluorescein diacetate
TBARS	Thiobarbituric acid reactive substances
CDNB	1-chloro2,4-dinitrobenzene
MDA	Malondialdehyde
PAHs	Polycyclic aromatic hydrocarbons
